# Complications and Revision Surgery in Orthopedics Focusing on Traumatology

**DOI:** 10.7759/cureus.65640

**Published:** 2024-07-29

**Authors:** Abdul Munaf Saud, Tauseef Raza, Muhammad Kamran, Muhammad Adeel, Syed Abdur Rub Abidi, Abdul Rehman Khan, Syed Taha Ahmed, Syed Muhammad Tayyab Hassan

**Affiliations:** 1 Orthopedic Surgery, Bahawal Victoria Hospital, Bahawalpur, PAK; 2 Orthopedics, KMU Institute of Medical Sciences, Kohat, PAK; 3 Orthopedics, Nishtar Medical University, Multan, PAK; 4 Orthopedics, Ayub Teaching Hospital, Ayub Medical College, Abbottabad, PAK; 5 Orthopedic Surgery, Jinnah Medical and Dental College, Karachi, PAK; 6 Orthopedics, Dow University Hospital, Dow International Medical College, Dow University of Health Sciences, Karachi, PAK; 7 Surgery, Sindh Medical College, Jinnah Sindh Medical University, Karachi, PAK; 8 Orthopedic Surgery, Dar As Sihha Medical Center, Dammam, SAU

**Keywords:** healthcare economics, surgical techniques, revision surgeries, complications, orthopedic traumatology

## Abstract

Background: Orthopedic traumatology, a vital component of orthopedic surgery, poses significant challenges in managing complications and necessitating revision surgeries. These challenges impact clinical outcomes, healthcare economics, and patient well-being.

Objective: This study aimed to provide insights that informed clinical decision-making and improved patient outcomes by thoroughly examining the range of complications encountered in orthopedic traumatology. Specifically, the research focused on the indications, techniques, and outcomes of revision surgeries.

Methodology: This retrospective cohort research looked at orthopedic traumatology complications and revision procedures over a thorough two-year period from March 2021 to March 2023 at Hayatabad Medical Complex in Peshawar, Pakistan. The following information was gathered from 316 patients receiving orthopedic surgery for traumatic injuries: demographics, kinds of trauma, surgical methods, complications, indications, methods, and results of revision surgery. For data analysis, chi-square tests and descriptive statistics were used, with the goal of finding patterns and correlations within the research population.

Results: The average age of the 316 patients was 42.5 years, and 64.76% of them were male (n = 192). The most frequent kind of trauma (n = 218; 69.01%) was fractures, which were mostly brought on by falls (n = 147; 46.52%). The most common surgical method (n = 138; 43.67%) was found to be internal fixation, which was followed by external fixation (n = 67; 21.20%). The most common complication (n = 78; 24.68%) was surgical site infection, which resulted in revision procedures mostly for infection (n = 68; 21.52%) and implant failure (n = 56; 17.72%). Debridement was the most often used revision approach (n = 95; 30.10%), and it was substantially correlated with surgical outcomes, such as increased function (31%) and full resolution (36%).

Conclusion: This research emphasizes the need to maximize patient outcomes for improved well-being and highlights the crucial role that careful care plays in managing complications and revision operations in orthopedic traumatology.

## Introduction

Orthopedic surgery, particularly in the realm of traumatology, represents a critical aspect of medical intervention aimed at restoring functionality and alleviating pain for patients suffering from musculoskeletal injuries [1]. The treatment of problems and the need for revision procedures are two major obstacles in this sector. These difficulties have an influence on clinical results in addition to costing healthcare systems money and maybe lowering patient quality of life overall [2,3]. Thus, for the purpose of maximizing patient care and resource allocation in healthcare settings, it is important to comprehend the complexities of complications and revision procedures in orthopedics, especially in traumatology [4].

Orthopedic surgery complications include a broad range of problems, from nerve damage and implant malfunctions to surgical site infections and bone misalignment [5,6]. Every problem offers a different therapeutic picture, necessitating early detection and effective care techniques to reduce unfavorable consequences [7]. Furthermore, the need for revision operations highlights the complexity inherent in orthopedic therapy, regardless of the reason for the need-complications or insufficient initial therapies [8]. Additional difficulties associated with revision operations include higher surgical risks, longer recovery times, and more psychological discomfort for patients [9].

In orthopedics, the frequency of problems and the need for revision procedures highlight the need for careful preoperative planning, exacting surgical methods, and conscientious postoperative care [10,11]. The chance of problems and the need for revision operations is greatly influenced by variables such as patient comorbidities, surgical technique, implant choice, and surgical competence [12]. Moreover, the field of orthopedic traumatology is constantly changing due to developments in surgical techniques and implant materials, which provide both possibilities and difficulties in terms of controlling complications and improving results [13].

The area of orthopedic traumatology is still dynamic, with continuing discussions and changing best practices, despite significant efforts being made to enhance surgical methods and lower the frequency of complications [14]. Therefore, for orthopedic surgeons, residents, and other healthcare providers engaged in the treatment of patients with musculoskeletal injuries, a thorough awareness of complications and revision operations is essential [15].

Research objective

This study aimed to provide insights that informed clinical decision-making and improved patient outcomes by thoroughly examining the range of complications encountered in orthopedic traumatology; specifically, the research focused on the indications, techniques, and outcomes of revision surgeries.

## Materials and methods

Study design and setting

This research was carried out in the Hayatabad Medical Complex in Peshawar, Pakistan, and used a retrospective cohort design. Retrospective cohort design made it possible to review historical patient data to evaluate the frequency of complications and the need for revision procedures in patients with orthopedic traumatology over a certain time frame. The trial period, which ran from March 2021 to March 2023, gave researchers a full two years to gather and analyze data.

Inclusion and exclusion criteria

During the study period, patients who received orthopedic surgery at Hayatabad Medical Complex for severe injuries met the inclusion criteria for this research. There were patients of all ages and genders present. Patients with non-traumatic orthopedic operations, insufficient medical data, congenital orthopedic problems, and non-orthopedic surgery recipients were among the exclusion criteria.

Sample size

A sample size of 316 patients was determined through power analysis, considering the estimated prevalence of complications and revision surgeries among orthopedic traumatology patients at Hayatabad Medical Complex.

Data collection

Data were gathered from the Hayatabad Medical Complex's medical records department. Relevant data was retrieved, such as the patient's demographics, the sort of trauma they had sustained, the surgical operations they had undergone, the problems they had, the reasons for the revision surgeries, the surgical methods they had used, and the postoperative results. Medical professionals with training collected the data to guarantee accuracy and consistency.

Statistical analysis

The research population's demographic and clinical data were compiled using descriptive statistics, including means, frequencies, percentages, and standard deviations. The study used chi-square tests to evaluate the correlation between category variables. At p < 0.05, statistical significance was established.

Ethical approval

The Institutional Review Board (IRB) at the Hayatabad Medical Complex in Peshawar, Pakistan, granted ethical clearance for this research. Throughout the data-gathering procedure, the research protocol protected patient privacy and confidentiality by adhering to the Declaration of Helsinki's tenets. Because the research was retrospective in nature, informed permission was not required, and patient data were anonymized to protect privacy.

## Results

The demographic distribution of the 316 patients receiving orthopedic therapy is shown in Table 1. With a standard variation of ±18.3 years, the patients' mean age is 42.5 years. The gender breakdown of the patients reveals that 124 are female (39.24%) and 192 are male (60.76%). Fractures account for 218 instances (69.01%) of all trauma categories. Dislocations come in second with 57 cases (18.04%) and soft tissue injuries with 25 cases (7.91%). Furthermore, 16 instances (5.05%) have trauma categories that fall under the "Others" group. According to mechanisms of injury, falls account for the majority of instances (47 cases, 46.52%), followed by car accidents, which account for 81 cases (25.63%), sports injuries, which account for 46 cases (14.56%), and other causes, which account for 42 cases (13.29%).

**Table 1:** Demographic characteristics of study population

Demographic characteristic		Mean ± SD/frequency (n = 316)	Percentage
Age in years	Mean ± SD	42.5 ± 18.3	-
Gender	Male	192	60.76
	Female	124	39.24
Type of trauma	Fracture	218	69.01
	Dislocation	57	18.04
	Soft tissue injury	25	7.91
	Others	16	5.05
Mechanism of injury	Fall	147	46.52
	Motor vehicle accident	81	25.63
	Sports injury	46	14.56
	Other	42	13.29

**Figure 1:**
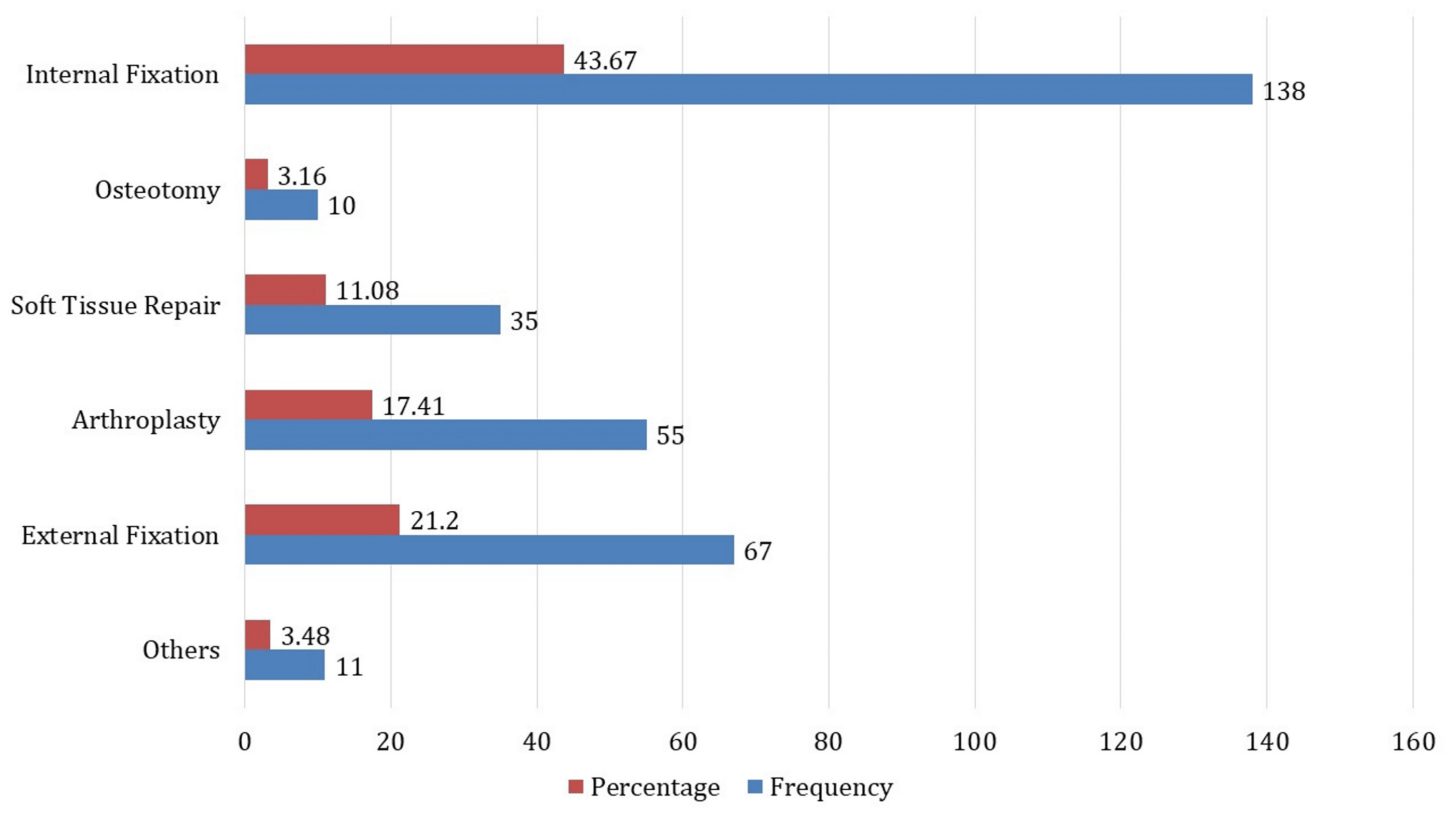
Distribution of surgical procedures

The distribution of surgical treatments among 316 patients receiving orthopedic interventions is shown in Figure 1. Of all the procedures, internal fixation is the most often performed, accounting for 138 instances or 43.67% of the total. Following with 67 instances, or 21.20% of operations, is external fixation. With 55 cases, or 17.41% of all surgeries, performed, arthroplasty ranks as the third most common operation. Of the treatments, 11.08% are reported in 35 instances where soft tissue healing is documented. Osteotomy is a less frequent operation, with 10 occurrences (3.16%) reported. Furthermore, a subcategory titled "Others" included less frequent operations, accounting for 3.48% of cases (n = 11).

**Figure 2:**
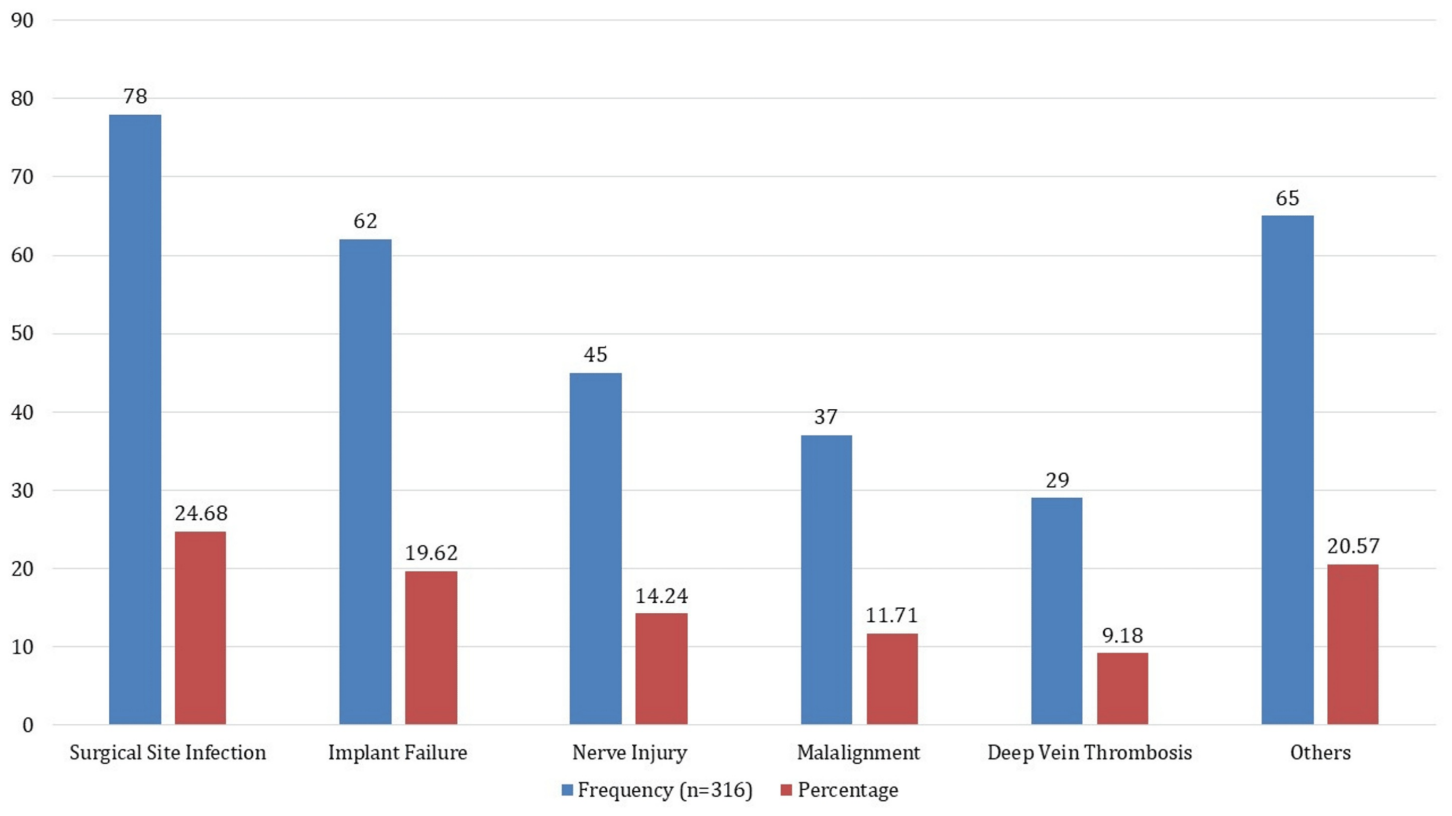
Complications encountered during orthopedic surgeries

Based on a sample size of 316 patients, Figure 2 breaks out the issues that arise following orthopedic procedures. Surgical site infection is the most common consequence, occurring in 78 instances and making up 24.68% of all sequelae. The next most common cause, with 62 occurrences (19.62%), is implant failure. Nerve damage ranks as the third most frequent consequence, occurring in 45 instances (14.24%). Thirty-nine patients (9.18%) had deep vein thrombosis, while 37 cases (11.71%) had malalignment. Furthermore, the "Others" group includes a variety of other difficulties, with a total of 65 instances (20.57%).

Table 2 presents the indications for orthopedic revision surgery based on 316 instances in the sample size. With 68 instances, or 21.52% of the total, surgical site infection is the most common indication. Implant failure comes in second, with 56 instances documented, or 17.72% of the total. The third most frequent indicator is malalignment, which is present in 41 instances (12.97%), whilst non-union or failure of union is noted in 34 cases (10.76%). In 27 instances, hardware irritation is reported (8.54%). Furthermore, the group "Others" includes a number of less frequent indicators, with 90 instances (28.48%) falling into this category. Furthermore, a p-value of 1.00 indicates that there is no meaningful correlation between the frequency of revision surgery and its indication.

**Table 2:** Indications for revision surgeries *: chi-square test; p-value < 0.05 is significant

Indication for revision surgery	Frequency (n = 316)	Percentage	p-value*
Surgical site infection	68	21.52	1.00
Implant failure	56	17.72	
Malalignment	41	12.97	
Non-union/failure of union	34	10.76	
Hardware irritation	27	8.54	
Others	90	28.48	

The frequencies and percentages of the several surgical procedures used for revision operations are shown in Table 3. With 30.10% of cases, the most often used procedure was debridement, which was followed by revision fixation (19.94%) and implant removal (17.72%). Soft tissue repair and bone grafting accounted for 9.18% and 16.46% of cases, respectively, and were also often used. In 8.54% of instances, other alternative methods were used. The statistical significance of the p-value, which is less than 0.05, indicates a noteworthy correlation between the surgical procedures used in revision operations.

**Table 3:** Surgical techniques employed for revision surgeries *: chi-square test; p-value < 0.05 is significant

Surgical technique	Frequency (n = 316)	Percentage	p-value*
Debridement	95	30.10	<0.05
Implant removal	56	17.72	
Revision fixation	63	19.94	
Bone grafting	52	16.46	
Soft tissue repair	29	9.18	
Others	27	8.54	

**Figure 3:**
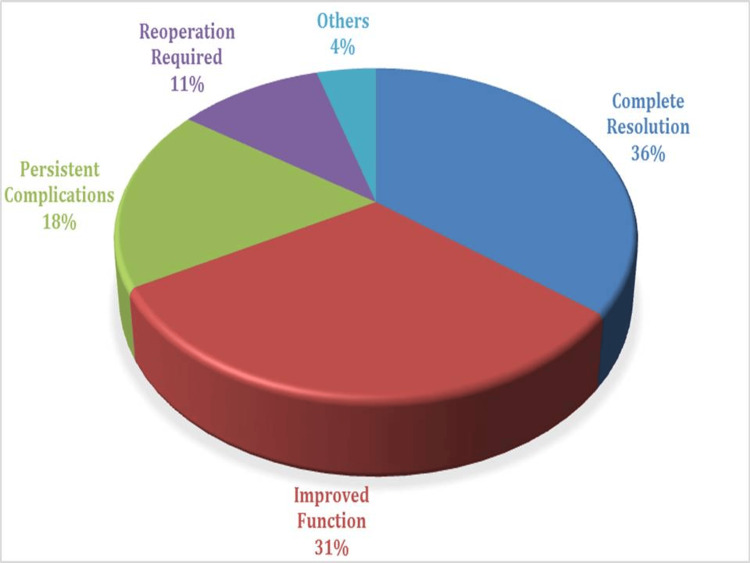
Postoperative outcomes of revision surgeries

The postoperative results of revision procedures are shown in Figure 3, which includes information on the frequency and percentage distribution of different outcomes in a sample of 316 patients. The most common result, "Complete Resolution," was 36% of instances, while "Improved Function" was 31%, quite close behind. In 18% of instances, there were "persistent complications," and in 11% of cases, "reoperation required." Also included in the category "Others" were results that were not specifically classified, accounting for 4% of instances.

The patient-reported outcomes reveal a range of satisfaction and pain levels (Table 4). Overall satisfaction was high, with 100 patients (31.65%) rating their experience as "Excellent" and 120 patients (37.97%) as "Good," while 70 patients (22.15%) considered their experience "Fair" and 20 patients (6.33%) "Poor." Pain levels, assessed using the visual analog scale (VAS), showed 100 patients (31.65%) reported "No Pain" with a mean score of 0.5 ± 0.7, 130 patients (41.51%) experienced "Mild Pain" with a mean score of 2.0 ± 1.0, 60 patients (18.99%) had "Moderate Pain" with a mean score of 4.0 ± 1.2, and 26 patients (8.23%) reported "Severe Pain" with a mean score of 6.5 ± 1.1. In terms of functional improvement, 120 patients (37.97%) reported "Significant Improvement" and another 120 patients (37.97%) "Moderate Improvement," whereas 50 patients (15.82%) experienced "Minimal Improvement" and 26 patients (8.23%) saw "No Improvement." Last, 180 patients (56.96%) were able to return to their pre-injury activities, while 136 patients (43.04%) were not.

**Table 4:** Patient-reported outcomes

Outcome category		Frequency (n = 316)	Percentage (%)	Mean score ± SD
Overall satisfaction	Excellent	100	31.65	-
	Good	120	37.97	-
	Fair	70	22.15	-
	Poor	20	6.33	-
	Not reported	6	1.90	-
Pain level (VAS score)	No pain	100	31.65	0.5 ± 0.7
	Mild pain	130	41.51	2.0 ± 1.0
	Moderate pain	60	18.99	4.0 ± 1.2
	Severe pain	26	8.23	6.5 ± 1.1
Functional improvement	Significant improvement	120	37.97	-
	Moderate improvement	120	37.97	-
	Minimal improvement	50	15.82	-
	No improvement	26	8.23	-
Return to pre-injury activity	Yes	180	56.96	-
	No	136	43.04	-

## Discussion

The study's findings provide a thorough grasp of orthopedic traumatology complications and revision operations, with noteworthy values recorded across a range of parameters. The average age of patients receiving orthopedic treatment in this study was 42.5 years, standard deviation of ±18.5. Additionally, the majority of trauma cases (69.01%) were fractures, which is in line with other studies that have shown fractures to be a primary indication for orthopedic interventions [16]. In a similar vein, the high frequency of falls as the primary cause of injury (46.52%) supports earlier research stressing the important role falls play in orthopedic trauma patients [17].

On surgical methods, the study's results on the prevalence of internal fixation (43.67%) are consistent with other studies highlighting the extensive use of internal fixation techniques in fracture care [18]. Furthermore, the high rate of implant failures (19.62%) and surgical site infections (24.68%) is consistent with data from related studies that have shown ongoing difficulties related to these complications in orthopedic procedures [19].

Similar patterns may be seen when comparing the indications for revision procedures with earlier studies. As an example, the most prevalent explanation in this study is surgical site infection (21.52%), which is in line with data from earlier research that showed infectious complications were a prominent cause of revision procedures in orthopedic traumatology [20]. In a similar vein, the frequency of implant failure as a reason for revision surgery (17.72%) is consistent with other research stressing how implant-related problems affect surgical results [21].

Furthermore, the distribution of surgical methods used in this study's revision operations corresponds to accepted standards in orthopedic treatment. The prevalence of debridement as a method that is often used (30.10%) is consistent with results from the literature that emphasize the role that debridement plays in wound care and infection prevention in orthopedic settings [22].

The overall consistency of the findings across many contexts and settings is highlighted by these comparisons with earlier research studies, which strengthens the validity and generalizability of the study's conclusions. This study advances our understanding of complications and revision procedures in orthopedic traumatology by expanding on previously held information and insights. This understanding will impact future research attempts and clinical practice.

Limitations

The study on complications and revision surgery in orthopedics provides valuable insights into managing traumatic injuries, but it is important to acknowledge its limitations for a thorough understanding of the findings. One potential limitation is the retrospective design of the research, which may introduce biases in data collection and analysis. Additionally, variability in surgical techniques among different surgeons could influence outcomes and add another layer of complexity to interpreting the results. Unmeasured confounding variables might also impact the study's findings, as not all potential influencing factors were controlled for. Despite these limitations, the study benefited from a substantial sample size of 316 patients, which enhances the robustness of the data. The research was conducted within a controlled environment, which allowed for a detailed exploration of complications and revision procedures. While the focused approach provides valuable insights, it may limit the generalizability of the findings to other healthcare settings. To address these limitations, efforts were made to standardize data collection and minimize biases, but further research with diverse settings and additional controls could offer a more comprehensive understanding of the study's strengths and weaknesses.

## Conclusions

The research highlights the substantial influence that complications and revision procedures have on patient care and healthcare systems, providing insight into the complex terrain of orthopedic traumatology. The results highlight the frequency of problems including implant failures and surgical site infections, which call for revision procedures when symptoms like non-union and malalignment occur. The research also emphasizes the need for careful surgical methods and postoperative management to reduce unfavorable results. This study adds to our knowledge of orthopedic traumatology by shedding light on the demographics, surgical techniques, and postoperative results. This helps inform clinical decision-making and direct future research efforts. Orthopedic traumatology is a dynamic discipline with a constant quest for excellence in patient care, as seen by the continual evolution of efforts to enhance patient outcomes.

## References

[1] Komasawa N (2024). Revitalizing postoperative pain management in enhanced recovery after surgery via inter-departmental collaboration toward precision medicine: a narrative review. Cureus.

[2] Taylor JM, Gropper MA (2006). Critical care challenges in orthopedic surgery patients. Crit Care Med.

[3] Kurtz SM, Lau EC, Ong KL, Adler EM, Kolisek FR, Manley MT (2017). Which clinical and patient factors influence the national economic burden of hospital readmissions after total joint arthroplasty?. Clin Orthop Relat Res.

[4] Choi J, Carlos G, Nassar AK, Knowlton LM, Spain DA (2021). The impact of trauma systems on patient outcomes. Curr Probl Surg.

[5] Stinson Z, Rosenfeld S, McNeil JC (2019). Infections complicating orthopedic surgery and implants. Healthcare-Associated Infections in Children.

[6] Bandopadhyay S, Bandyopadhyay N, Ahmed S, Yadav V, Tekade RK (2019). Current research perspectives of orthopedic implant materials. Biomater Bionanotechnol.

[7] Lebude B, Yadla S, Albert T (2010). Defining "complications" in spine surgery: neurosurgery and orthopedic spine surgeons' survey. J Spinal Disord Tech.

[8] Dailey EA, Cizik A, Kasten J, Chapman JR, Lee MJ (2013). Risk factors for readmission of orthopaedic surgical patients. J Bone Joint Surg Am.

[9] Palmer CK, Gooberman-Hill R, Blom AW, Whitehouse MR, Moore AJ (2020). Post-surgery and recovery experiences following one- and two-stage revision for prosthetic joint infection - a qualitative study of patients' experiences. PLoS One.

[10] Koormala M, Choalla N, CH IP, Rathod PS (2024). Evaluation of perioperative complications in patients with obstructive sleep apnea: an observational analysis with clinical implications. Asian J Med Sci.

[11] Cursaru A, Popa MI, Cretu B (2023). Insights from a comprehensive case series analysis: exploring individualized approaches to managing Vancouver B periprosthetic femoral fractures. Orthop Sports Med.

[12] Schoenfeld AJ, Carey PA, Cleveland AW 3rd, Bader JO, Bono CM (2013). Patient factors, comorbidities, and surgical characteristics that increase mortality and complication risk after spinal arthrodesis: a prognostic study based on 5,887 patients. Spine J.

[13] Liang W, Zhou C, Bai J (2024). Current advancements in therapeutic approaches in orthopedic surgery: a review of recent trends. Front Bioeng Biotechnol.

[14] Conway DJ, Coughlin R, Caldwell A, Shearer D (2017). The institute for global orthopedics and traumatology: a model for academic collaboration in orthopedic surgery. Front Public Health.

[15] Epstein S, Sparer EH, Tran BN, Ruan QZ, Dennerlein JT, Singhal D, Lee BT (2018). Prevalence of work-related musculoskeletal disorders among surgeons and interventionalists: a systematic review and meta-analysis. JAMA Surg.

[16] Meling T, Harboe K, Søreide K (2009). Incidence of traumatic long-bone fractures requiring in-hospital management: a prospective age- and gender-specific analysis of 4890 fractures. Injury.

[17] Zhu Y, Chen W, Xin X (2020). Epidemiologic characteristics of traumatic fractures in elderly patients during the outbreak of coronavirus disease 2019 in China. Int Orthop.

[18] Lee C, Pereira C, Zoller S (20191). Feasibility and reliability of open reduction internal fixation in delayed distal radius fracture management. J Hand Surg Glob Online.

[19] Campoccia D, Montanaro L, Arciola CR (2006). The significance of infection related to orthopedic devices and issues of antibiotic resistance. Biomaterials.

[20] Heinz NR, Clement ND, Young RN, Duckworth AD, White TO, Molyneux SG (2023). Rate and factors associated with surgical site infection following aseptic revision fixation of orthopaedic trauma injuries. Eur J Orthop Surg Traumatol.

[21] Khan M, Osman K, Green G, Haddad FS (2016). The epidemiology of failure in total knee arthroplasty: avoiding your next revision. Bone Joint J.

[22] Attinger CE, Janis JE, Steinberg J, Schwartz J, Al-Attar A, Couch K (2006). Clinical approach to wounds: débridement and wound bed preparation including the use of dressings and wound-healing adjuvants. Plast Reconstr Surg.

